# How accurate are autistic adults and those high in autistic traits at making face-to-face line-of-sight judgements?

**DOI:** 10.1177/1362361320909176

**Published:** 2020-03-13

**Authors:** Megan Freeth, Emma Morgan, Patricia Bugembe, Aaron Brown

**Affiliations:** University of Sheffield, UK

**Keywords:** autism, autistic traits, ecological validity, gaze following, line-of-sight judgements, social cognition and social behaviour

## Abstract

Being able to follow the direction of another person’s line-of-sight facilitates social communication. To date, much research on the processes involved in social communication has been conducted using computer-based tasks that lack ecological validity. The current paradigm assesses how accurately participants can follow a social partner’s line-of-sight in a face-to-face scenario. In Study 1, autistic and neurotypical adults were asked to identify which location, on a grid of 36 potential locations, the experimenter was looking at on a series of discrete trials. All participants (both autistic and neurotypical) were able to effectively make line-of-sight judgements, scoring significantly above chance. Participants were also just as effective at making these judgements from either a brief, 1s, glance or from a prolonged, 5s, stare. However, at the group level, autistic participants were significantly less accurate than neurotypical participants overall. In Study 2, potential variation in performance along the broad autism phenotype was considered using the same paradigm. Bayesian analyses demonstrated that line-of-sight judgement accuracy was not related to the amount of autistic traits. Overall, these findings advance the understanding of the mechanistic processes of social communication in relation to autism and autistic traits in a face-to-face setting.

**Lay abstract:**

In order to effectively understand and consider what others are talking about, we sometimes need to follow their line-of-sight to the location at which they are looking, as this can provide important contextual information regarding what they are saying. If we are not able to follow other people’s line-of-sight, this could result in social communication difficulties. Here we tested how effectively autistic and neurotypical adults are at following a social partner’s line-of-sight during a face-to-face task. In a first study, completed by 14 autistic adult participants of average to above-average verbal ability and 14 neurotypical adult participants, we found that all participants were able to effectively follow the social partner’s line-of-sight. We also found that participants tended to be as effective at making these judgements from both a brief, 1s, glance or a long, 5s, stare. However, autistic adults were less accurate, on average, than neurotypical adults overall. In a second study, a separate group of 65 neurotypical adults completed the same line-of-sight judgement task to investigate whether task performance was related to individual variation in self-reported autistic traits. This found that the amount of self-reported autistic traits was not at all related to people’s ability to accurately make line-of-sight judgements. This research isolates and furthers our understanding of an important component part of the social communication process and assesses it in a real-world context.

## Introduction

Being able to follow the gaze of a social partner is a skill fundamental to effective social communication. The target location of a person’s gaze often provides important information, such as indicating their desires and intentions, or may correspond to an important aspect of the environment (Ristic et al., 2005). The gaze direction of a social partner is such a captivating cue that we tend to spontaneously follow it (Langton & Bruce, 1999; Senju et al., 2008) even if gaze direction is not predictive of anything (Driver et al., 1999). Effective gaze following facilitates the development of joint attention (Mundy & Newell, 2007). This, in turn, contributes to other communicative skills such as language acquisition (Adamson et al., 2009; Brooks & Meltzoff, 2005) and theory of mind development (Baron-Cohen, 1995; Charman et al., 2000).

For individuals with a diagnosis on the autism spectrum, the development of joint attention does not follow the typical trajectory (e.g. Dawson et al., 1998, 2004; Gillespie-Lynch et al., 2013; Leekam et al., 2000; Vivanti et al., 2014). It has clearly been demonstrated that infants and children with an autism diagnosis do not process and utilise gaze cues as effectively as their typically developing peers (Birmingham et al., 2017; Goldberg et al., 2008; Stauder et al., 2011), and the extent of the difficulties predicts symptom severity and later outcomes (Ibañez et al., 2013; Mundy et al., 1990; Sigman & Ruskin, 1999). It is proposed that humans have a specific neurocognitive system dedicated to eye direction detection and that autistic individuals experience difficulties with this (Baron-Cohen, 1995). However, the exact subcomponents of gaze processing that contribute to these difficulties are yet to be determined (Palanica & Itier, 2011).

It has previously been suggested that autistic individuals lack the ability to accurately follow eye gaze direction during naturalistic interactions (Leekam et al., 2000), although impairments in gaze direction detection do not always correspond with impairments in visual perspective taking (Leekam et al., 1997). This suggests that children with a diagnosis of autism rely on the presence of objects in their visual field to guide attention during naturalistic interactions. By contrast, evidence from computer-based studies with autistic individuals has been equivocal; a number of studies have reported difficulties with making line-of-sight judgements when several visual distractors are present (Rombough & Iarocci, 2013), while other studies report spontaneous, accurate gaze following in response to complex static scenes (Freeth et al., 2010a, 2010b; Sheth et al., 2011).

A recent study by Pantelis and Kennedy (2017) suggests that fine-grained line-of-sight judgements are made with reduced consistency and accuracy in autistic compared to neurotypical adults. There is also a general tendency for gaze direction judgements to be biased towards being more direct than is actually the case, with this effect being evident to a similar extent in both autistic and neurotypical adults (Pell et al., 2016). However, to date, these specific aspects of line-of-sight judgements have not been assessed in a face-to-face setting. When attempting to understand the mechanisms of social communication, it is important to study phenomena not only via computer-based tasks but also via scenarios when the social partner is physically present. This is important as qualitatively and quantitatively different effects can occur in live interactions compared to tasks where there is no social partner physically present (Freeth et al., 2013; Laidlaw et al., 2011; Risko et al., 2012). These differences have been suggested to arise due to the dual nature of gaze, with eyes capable of both communicating and receiving information – a critical characteristic which is absent when viewing others via a pre-recorded stimulus on a computer screen (Risko et al., 2016). It is therefore necessary to use naturalistic stimuli in order to determine if results found in isolated lab-based environments are likely to generalise into real-world settings (Risko et al., 2012).

Many important insights into autistic social attention have emerged from naturalistic interaction studies, though such studies tend not to have the capacity to pinpoint whether specific aspects of gaze following are impaired or problematic (Birmingham et al., 2017). Important factors that have the capacity to influence gaze following include motivation to attend to social stimuli, finding eyes or faces aversive, initiating or responding to joint attention bids, inference of social meaning, detection of direct versus averted gaze, and the ability to accurately make line-of-sight judgements. It is therefore yet to be determined what subcomponents of gaze direction detection are implicated in the atypicalities often evident in autism spectrum conditions (d’Arc et al., 2017). Studies either tend to isolate a specific component of gaze following, but without an ecologically valid social context, or to improve the understanding of gaze following behaviour within an ecologically valid social context, but without the possibility of isolating component processes. A study that began to address this limitation was conducted by Lachat et al. (2012) which investigated whether the gaze cueing effect (GCE) occurs in face-to-face situations, as has been observed in computer-based tasks. The GCE is the tendency for participant attention to be shifted to a gazed at location even when the direction of gaze is not related to task goals. Lachat et al.’s (2012) findings indicated that their face-to-face paradigm did indeed elicit the GCE. The authors note that further ecologically valid paradigms that isolate specific aspects of the gaze following process are needed to build an ecologically valid model of social communication. Here we address this gap by developing a paradigm to isolate one aspect of the gaze following process embedded within an ecologically valid context – the ability to accurately follow a social partner’s line-of-sight, assessed during a face-to-face interaction.

In real-world interactions, gaze cues can sometimes involve a prolonged stare at a target location and at other times involve only a brief glance. There is a broad range of evidence to suggest that, for autistic individuals, impairments of visual disengagement are evident from infancy to adulthood (see Sacrey et al., 2014, for a review). Landry and Parker (2013) collated evidence demonstrating that, in general, autistic individuals struggle when task requirements necessitate rapid attention orientation shifts. Furthermore, they speculate that slowing down the pace of social interactions may be beneficial for autistic individuals to enable them to ‘keep up’ with interactions. However, few studies have specifically investigated whether the duration of a gaze cue has a differential effect on gaze following in autistic compared to neurotypical individuals. One study that systematically investigated whether altering the cue-target stimulus-onset asynchrony had a differential effect on autistic compared to neurotypical children was conducted by Pruett et al. (2011). Although there were trends for between-group differences, no statistical differences between groups were observed. Given limitations of study power in relation to this particular question of between-group differences in this study, there was no strong evidence to accept the null hypothesis. Hence, the study findings in relation to potentially differential between-group effects in relation to gaze cue timings were therefore ambiguous. To our knowledge, no studies have investigated whether autistic individuals find it particularly difficult to judge a social partner’s line-of-sight from a brief glance. The current paradigm was therefore designed to answer this question.

Some difficulties experienced by autistic individuals are often also evident in individuals who do not meet the diagnostic criteria for autism but do express high levels of autistic behavioural traits, known as the broad autism phenotype (BAP). For example, individuals with no clinical diagnosis on the autism spectrum but who are high in autistic behavioural traits display differences from those low in autistic behavioural traits on measures of perception and cognitive function (Almeida et al., 2010; Brock et al., 2011; Grinter et al., 2009), social cognition (Sasson et al., 2013) and social attention (Chen & Yoon, 2011; Freeth et al., 2013; Vabalas & Freeth, 2016), though difficulties tend to be less pronounced in BAP individuals compared to those with a clinical diagnosis of autism. Here we present a novel paradigm assessing line-of-sight judgement accuracy in a face-to-face interaction in two separate cohorts of participants. Participants in Study 1 were from two distinct groups: autistic adults and age-, gender- and ability-matched neurotypical participants. Participants in Study 2 were a sample of university students whose behavioural traits were assessed using the Broad Autism Phenotype Questionnaire (BAPQ; Hurley et al., 2007). The paradigm required participants to judge which location on a grid, placed between the experimenter and the participant, the experimenter was looking at on a series of discrete trials. If a critical reason why autistic individuals tend to follow the gaze direction of others less than neurotypical individuals is due to reduced accuracy in making line-of-sight judgements, then poorer task performance will be observed. However, if the ability to make line-of-sight judgements is intact, and difficulties are in other areas (e.g. social motivation, eye aversion, initiating or responding to joint attention bids, inferring social meaning), then comparable performance in autistic and neurotypical individuals on this task will be observed. Trials were presented to participants in two main blocks: trials in one block involved the experimenter directing a prolonged stare to a grid location on each trial (gaze cue duration of 5 s per trial) and the other block involved the experimenter directing a brief glance to a grid location on each trial (gaze cue duration of 1 s per trial). If, as suggested by Landry and Parker (2013), it is the requirement to rapidly shift attention that is particularly problematic for autistic individuals in social interactions, trials that only present a brief glance to the target location will result in particularly poor performance by autistic individuals compared to neurotypical individuals. Whether higher BAPQ scores are associated with reduced line-of-sight judgements and whether higher BAPQ scores are more strongly correlated with performance accuracy in the brief glance trials compared to the prolonged stare trials will also be investigated. Previous research has indicated that the latency of gaze shifts is related to verbal intelligence (Falck-Ytter et al., 2012), and therefore participants were also asked to complete a measure of verbal IQ in order to ensure that differences in task performance could not be explained by variance in the participants’ verbal abilities.

## Study 1: how accurate are autistic adults at gaze following in face-to-face interactions

### Method

#### Participants

In total, 14 autistic adults (11 male and 3 female) and 14 neurotypical adults (11 male and 3 female) participated in this study. Participants were matched one to one on gender, age (within 5 years) and verbal IQ, assessed using the Wechsler Abbreviated Scale of Intelligence (WASI). All participants on the autism spectrum had received an official diagnosis from a clinical psychologist in the United Kingdom based on *Diagnostic and Statistical Manual of Mental Disorders* (*DSM*) or International Classification of Diseases (ICD) criteria. All participants also completed the BAPQ (Hurley et al., 2007). The questionnaire features a cut-off point of 108 (with those scoring above this cut-off classified as having a BAP) and three additional subscales of measurement (Aloof, Rigid and Pragmatic Language). The BAPQ has demonstrated a high sensitivity (>70%) to detecting these phenotypes and therefore was suitable for use in this study to provide an indication of current behavioural traits associated with the autism phenotype. An independent-samples *t* test indicated a highly significant difference between groups on total BAPQ score as the autistic participants scored much higher than the neurotypical participants, *t*(26) = 4.56, *p* < 0.001, *d* = 1.79 (see Table 1). Ethical approval for this study was obtained from the University of Sheffield Department of Psychology Ethics Sub-committee. All participants provided written informed consent prior to beginning the testing session.

**Table 1.** Participant characteristics.

	Autism participants	Neurotypical participants
No. of participants (male; female)	14 (11; 3)	14 (11; 3)
Age		
Mean	37.4	35.7
SD	13.3	13.6
Range	22–57	19–57
Verbal IQ		
Mean	112.6	115.3
SD	12.6	8.6
Range	88–128	100–136
BAPQ		
Mean	136.7**	96.0**
SD	25.3	21.8
Range	89–185	62–137

SD: standard deviation; BAPQ: Broad Autism Phenotype Questionnaire.

** *p* < 0.001 (difference between groups).

#### Design

The study used a mixed-measures design. There was a within-subject factor of gaze cue duration (1 vs 5 s) and a between-subject factor of group (autistic vs neurotypical). The dependent measure was line-of-sight judgement mean error score, providing an overall measure of accuracy for each participant. Error score was determined on each trial by calculating the number of grid locations horizontally and vertically, between the cued location and the response location. The horizontal and vertical error scores were then converted to an overall trial error score using Pythagoras’ theorem to determine gaze following accuracy. For example, an error of one grid location horizontally and three grid locations vertically would give an error score of 3.16, that is, √(1^2^ + 3^2^). The mean error score across all trials was then calculated for each participant. Higher mean error scores indicate reduced accuracy in line-of-sight judgements.

#### Procedure

**Figure 1.**
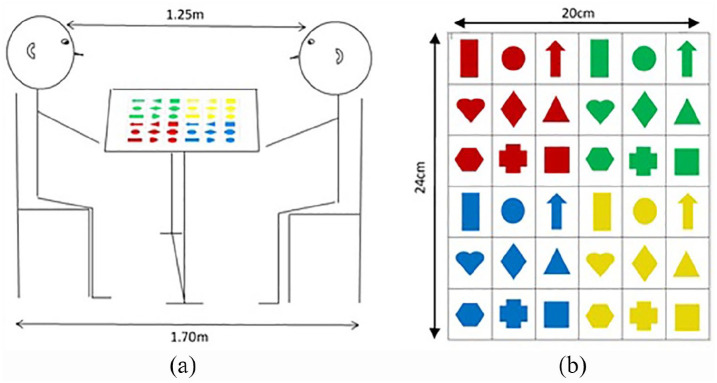
(a) Diagram of the experimental set-up and (b) the stimulus grid used within the experiment.

The participant and experimenter sat on chairs either side of a table; the back legs of the chairs were 1.70 m apart, resulting in the distance between the experimenter’s eyes and the participant’s eyes being approximately 1.25 m. Participants were informed that their back should make contact with the chair back throughout the experiment. All participants were tested by the third author. A stimulus grid was laid flat in the centre of the table (Figure 1(a)). The stimulus grid comprised a 6 × 6 location grid, that is, 36 potential target locations, each of which contained a coloured shape (Figure 1(b)). Each grid location measured 3.9 cm vertically and 3.3 cm horizontally and the coloured shapes measured on average 2.3 cm high and 1.9 cm wide. Therefore, each grid location subtended an approximate visual angle of 1.8° × 3.0°. In all testing sessions, the experimenter wore a plain dark-coloured top and kept the same hairstyle to ensure that the visual array was consistent between participants.

Participants were informed that they were to take part in a gaze following task that involved identifying which target location they believed the experimenter had looked at on a series of individual trials. Participants completed two blocks of 30 trials which included a short break after every 10 trials. Trials in Block A involved the experimenter looking at a particular target location for 5 s per trial. In Block B, the experimenter looked at each target location for 1 s per trial. Block order was counterbalanced between participants. All participants completed 10 practice trials prior to the main testing blocks to enable them to become familiar with the procedure. During the practice trials, feedback on performance accuracy was provided and the correct location was indicated by the experimenter on each trial. An audio-recording was used to ensure that every trial in each condition was accurately paced. The audio-recording prompted ‘Ready’, which triggered the experimenter to look directly at the participant’s face. There was then a pause of 1 s. The recording then prompted ‘Now’, which triggered the experimenter to direct her gaze to a target shape as determined by a trial order list sheet held by the experimenter. The trial order list sheet was the same for each participant. Gaze cues were terminated by a ‘BEEP’ after the designated cue time had elapsed. This triggered the experimenter to look up to the participant’s face. The participant was then required to point to the grid location at which they thought the experimenter had been looking. There was a gap of 8 s between trials to allow the participant to respond and the experimenter to record the grid location indicated by the participant.

This task was completed as part of a battery of tests, others of which are reported elsewhere (Freeth & Bugembe, 2019). The BAPQ and WASI were also completed during the same testing session as the line-of-sight judgement task.

### Results

**Figure 2.**
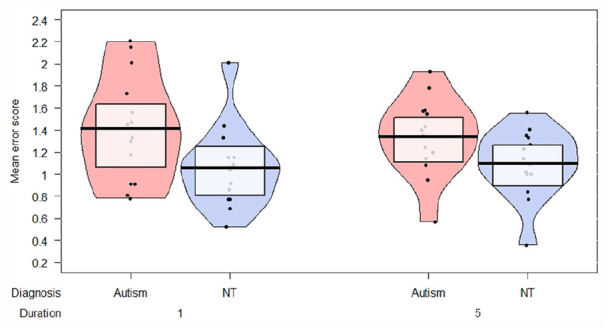
Mean error scores of line-of-sight judgements for autistic and neurotypical participants. Horizontal bars represent group means; shaded boxes represent 95% confidence intervals. Chance responding would elicit a mean error score of approximately 2.75.

Random, or chance, responding throughout the task would have resulted in overall mean error scores being not significantly better than (i.e. below) 2.75. This figure was determined based on a simulation of 100 datasets, where the responses for each trial were random numbers generated between 1 and 6 in order to simulate chance performance. Error scores were then calculated for these data. One-sample *t* tests revealed that participants in both groups performed significantly better than chance: autism group – *t*(13) = 13.81, *p* < 0.001, *d* = 7.65; neurotypical group – *t*(13) = 22.7, *p* < 0.001, *d* = 12.58 (see Figure 2). Indeed, inspection of the data revealed that every participant tested scored better than chance, that is, below 2.75 overall.

Potential difference in performance between the groups was then assessed using a 2 × 2 (group × trial duration) mixed-measures analysis of variance (ANOVA). This revealed a main effect of group, *F*(1, 26) = 5.77, *p* = 0.024, $\eta_{p}^{2}=0.18$, indicating that the autistic participants were significantly worse at the task than the neurotypical participants (mean error score autism = 1.38, 95% confidence interval (CI) = 1.16–1.59; mean error score neurotypical = 1.08, 95% CI = 0.92–1.24). There were no main effect of trial duration, *F*(1, 26) = 0.52, *p* = 0.82, $\eta_{p}^{2}=0.002$, and no interaction between group and trial duration, *F*(1, 26) = 0.56, *p* = 0.46, $\eta_{p}^{2}=0.021$, indicating that participants in neither group found the brief glance gaze cue (1s) trials more difficult than the prolonged stare gaze cue (5s) trials.

**Figure 3.**
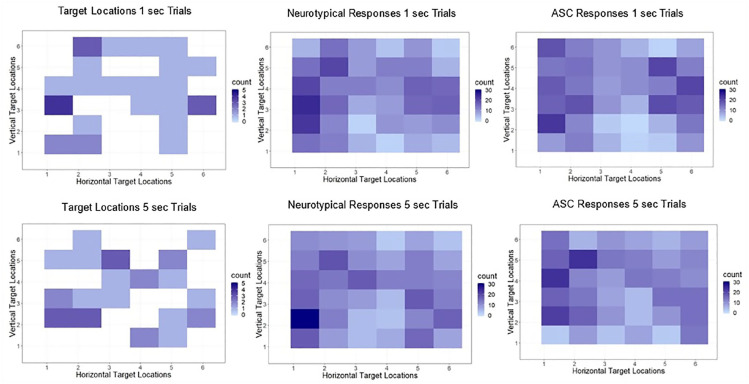
Overview of the spatial location of the target, the spatial location of the neurotypical participants’ responses and the spatial location of the autistic participants’ responses in both 1- and 5-s gaze cue conditions.

In order to determine whether either group was biased to perceive the interviewer’s eye gaze as directed towards themselves, analyses were conducted on the frequency of errors made towards the midline of the stimulus grid. The total number of trials where target locations were in Columns 1, 2, 5 or 6 and where the participant response was biased towards the midline was calculated for each participant. These totals were then compared between groups using independent-samples *t* tests to investigate whether one group made more centrally biased responses than the other. For the 1-s trials, neither group was more likely than the other to make centrally biased responses, *t*(26) = 1.22, *p* = 0.23 (autism – mean = 5.71, standard deviation (SD) = 3.15; neurotypical – mean = 4.36, SD = 2.73). There was also no difference between groups for the 5-s trials, *t*(26) = 0.75, *p* = 0.46 (autism – mean = 4.29, SD = 3.07; neurotypical – mean = 3.43, SD = 3.01) (see Figure 3). Therefore, neither group was more likely to perceive the experimenter’s eye gaze as being directed towards themselves.

## Study 2: does the level of autistic traits predict face-to-face gaze following accuracy

### Method

#### Participants

A total of 69 18- to 23-year-old student participants (29 males and 40 females) completed this study. Verbal IQ was assessed using the WASI. All participants completed the BAPQ. Two participants self-reported having an anxiety disorder and three participants self-reported having an autism diagnosis and so were excluded from the analyses. See Table 2 for details of the final participant cohort.

**Table 2.** Participant characteristics.

	Participants
No. of participants (male; female)	64 (25; 39)
Age	
Mean	19.7
SD	1.5
Range	18–23
Verbal IQ	
Mean	118.4
SD	14.9
Range	80–144
BAPQ	
Mean	92.9
SD	20.7
Range	55–149

SD: standard deviation; BAPQ: Broad Autism Phenotype Questionnaire.

#### Design and procedure

The design and procedure was the same as those for Study 1, except that the participants were not categorised into diagnostic groups, rather an individual’s autistic traits score (BAPQ score) was considered a continuous measure. Also, the within-subject factor of gaze cue duration (1 vs 5 s) included more data as each block contained *n* = 50 trials. It was possible to increase the number of trials in Study 2 compared to Study 1 as this was the only experimental task being completed by participants in this testing session so we were less concerned about participant fatigue. Participants were either tested by the first author (M.F.) (*n* = 39) or the fourth author (A.B.) (*n* = 30).

### Results

In order to check that participant characteristics were similar between participants tested by M.F. and those by A.B., an independent-samples *t* test was conducted on BAPQ scores indicating no between-group difference, *t*(62) = 1.44, *p* = 0.15.

Mean error scores for all participants in both the prolonged stare trials (5s gaze cue per trial) and brief glance trials (1-s gaze cue per trial) were calculated. Mean error scores for the 5s trials were lower than those for the 1s trials, *t*(63) = 4.23, *p* < 0.001, *d* = 0.32 (mean error score for 5s trials = 1.11; mean error score for 1s trials = 1.23) indicating better performance on the trials where the gaze cue was presented for longer; this was a small–medium sized effect.

**Figure 4.**
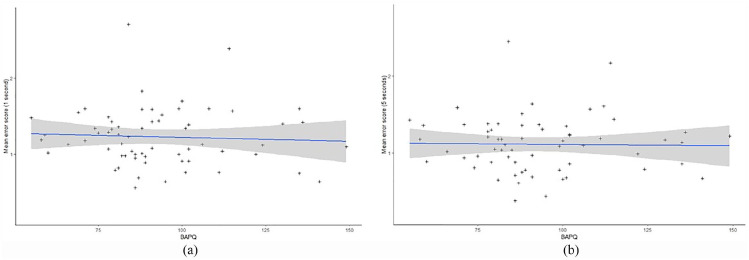
(a) The relationship between autistic traits and performance on brief glance (1-s) gaze cue trials. Blue line indicates line of best fit, and shaded area indicates 95% confidence region. (b) The relationship between autistic traits and performance on prolonged stare (5-s) gaze cue trials. Blue colour indicates 95% confidence region.

In order to address the main research question of whether higher autistic traits were associated with poorer performance on line-of-sight judgements, a Pearson bivariate correlation between mean error score and BAPQ score was conducted. No significant correlation between autistic traits and mean error scores was observed on either the 5s cue trials, *r* = –0.02, *p* = 0.89, or the 1s cue trials, *r* = –0.06, *p* = 0.66. Furthermore, there was no significant relationship between mean error scores and any of the BAPQ subscales for either the 1-s (*p* > 0.05) or the 5-s (*p* > 0.05) trials. Bayesian analyses^1^ confirmed that there was strong support for the null hypothesis for both the 1-s cue trials, BF_H0_ = 5.81, and the 5s cue trials, BF_H0_ = 6.35, clearly demonstrating that there was no association between line-of-sight judgement accuracy and autistic traits (see Figure 4). There were also no significant relationships between mean error scores and BAPQ subscale scores (all *p*s = ns) suggesting that making line-of-sight judgements in a naturalistic setting is not more difficult for individuals who are high in autistic traits.

Due to finding that, in this study, line-of-sight judgements were more accurate in the 5s trials compared to the 1s trials, the additional benefit of a longer gaze cue was assessed by calculating the difference in performance between the 1s and the 5s trials. There was no significant correlation between autistic traits and the magnitude of improved performance between the 5s and the 1s trials (*r* = –0.07, *p* = 0.58), indicating that particular difficulty making line-of-sight judgements from a brief glance compared to a prolonged state was not associated with the amount of autistic traits.

## General discussion

The aim of the two studies presented was to investigate whether autistic adults are as accurate as neurotypical adults at making face-to-face line-of-sight judgements and whether accuracy of line-of-sight judgements is related to autistic traits. The findings demonstrated that all participants tested were able to follow a social partner’s line-of-sight at above chance levels from either a brief glance (1 s per trial) or a prolonged stare (5 s per trial). However, Study 1 found that, at the group level, autistic adults were less accurate overall compared to neurotypical participants indicating that this particular aspect of social communication does tend to be difficult for autistic adults; this was a medium–large effect. This therefore suggests that although autistic participants are able to make line-of-sight judgements there is a certain degree of increased difficulty when doing so compared to neurotypical controls, though the underlying reasons for this remain to be determined.

It is important to note that a minority of autistic participants did not display any difficulties with the task indicating heterogeneity within the group of autistic adults in relation to this particular skill, indeed some autistic participants performed better than the average neurotypical participant. Study 1 also demonstrated that autistic adults were able to make line-of-sight judgements as accurately when the gaze cue was a brief glance (1 s per trial) compared to when it was a prolonged stare (5 s per trial), thus demonstrating that difficulties with social communication are unlikely to be due to gaze cues only being presented briefly in social interactions, though we do acknowledge that the relatively small sample size reported here does not allow us to detect small effects. However, the results indicate that the cause of difficulties is likely derived from other aspects of social communication. Study 2 found no relationship between line-of-sight judgement accuracy and autistic traits (as assessed via the BAPQ), indicating that this skill is not one of the areas of difficulty of social communication associated with autistic traits within individuals without a clinical diagnosis of autism. In contrast to the findings of Study 1, in Study 2, somewhat poorer performance on brief glance trials was observed compared to prolonged stare trials; this was a small–medium sized effect. However, the magnitude of the difference in performance between the trials types was not associated with autistic traits, indicating no evidence that autistic traits play a role in how effectively an individual is able to make face-to-face line-of-sight judgements.

It is encouraging to observe that all autistic adults tested were able to follow brief gaze cues demonstrating that this is not an area of major deficit for autistic adults of average to above-average ability, although it is important to note that even small deficits in an ability can lead to difficulties in everyday life. It has previously been suggested that autistic children require specific objects to be located in the visual array in order for gaze direction to be followed (Leekam et al., 2000); however, other research has suggested that gaze impairments in autistic children decrease with age (Webster & Potter, 2008). This study demonstrated that autistic adults did not require objects to be present in order for gaze to be followed. Indeed, the task presented 36 different potential grid locations to participants which each could have been the target location on each trial, resulting in a very challenging task. All participants were able to perform well above chance levels, though, in accordance with the adulthood findings of Pantelis and Kennedy (2017), the line-of-sight judgements made by autistic adults were not quite as fine-grained as in neurotypical adults. Whereas the study by Pantelis and Kennedy (2017) used a computer-based task, our study is the first to demonstrate that, when compared to neurotypical adults, the line-of-sight judgements of autistic adults tend not to be quite as fine-grained in a face-to-face interaction.

This study successfully isolated and assessed a specific component of social communication. However, many of the other skills that successful free-flowing social interactions typically require were absent (e.g. motivation to attend to social stimuli; selectively attending to the eyes or face; spontaneously initiating or responding to joint attention bids; inferring social meaning). This provided insight into the performance of the component process in question and therefore contributes to the development of a mechanistic model of social communication in autistic adults. An important future direction will be for other component processes to be isolated and tested during face-to-face interactions so that specific areas of strength and difficulty can be identified. Work already conducted by Lachat et al. (2012) that contributes to this process demonstrated that the GCE is evident in face-to-face interactions. In addition, our previous work on social attention in a face-to-face conversation demonstrated that autistic adults tend to avert their gaze away from the social partner a lot more than neurotypical adults do when the social partner attempts to make direct eye contact, resulting in reduced opportunities for reciprocal social gaze (Freeth & Bugembe, 2019), but further work on other component processes is now required in order to build a model of autistic naturalistic social communication.

This study did not find evidence to support Landry and Parker’s (2013) suggestion that slowing down the pace of a social interaction could be beneficial for autistic individuals to enable them to ‘keep up’ with interactions. Autistic participants in this study were just as able to make line-of-sight judgements when the gaze cue was presented as a brief glance compared to a prolonged stare. However, it may well be that prolonging the presentation of other aspects of social communication information may be beneficial to autistic adults. It could also be that the simplicity of this task and unambiguous nature of task instructions, or indeed the presentation of a single piece of social information on each individual trial rather than the presentation of multiple cues, facilitated performance. It has previously been shown that increased social complexity, when presenting computer-based stimuli, results in clearer differences between the social attention of autistic and neurotypical individuals being observed (Chevallier et al., 2015). The extent to which increasing social complexity has an effect on performance in face-to-face interactions is yet to be determined. These will be questions for future research.

The skill of being able to accurately follow another person’s line-of-sight to a specific target location is clearly a skill that has the potential to assist inferences about communicative intent. The ability to direct attention to social cues is thought to aid in understanding the actions of others (Loucks & Sommerville, 2013). As the ability to make line-of-sight judgements was somewhat reduced in autistic adults compared to neurotypical adults, it is important to consider that this may subsequently impact the ability of autistic individuals to generate inferences about the preferences and intentions of their social partners. There is therefore potentially some scope for improvement in this ability. It may be that some additional practice with gaze following would improve this skill. Targeting improvements in joint attention in childhood has been the focus of many social skills intervention studies (e.g. Kasari et al., 2006; Murza et al., 2016). However, it is yet to be clearly determined whether training such skills has a positive effect on long-term outcomes such as friendships (Freeman et al., 2015) or whether the result is merely that individuals are being trained to appear more neurotypical without any resulting long-term tangible benefit to the individual. Therefore, we would be reticent to recommend training of gaze following accuracy in adulthood as a skill in itself.

Study 2 enabled a sensitive investigation into whether an individual’s behaviour traits associated with the BAP predict line-of-sight following accuracy. The study findings clearly demonstrated that there was no relationship between autistic traits and line-of-sight following accuracy. Strong evidence in support of the null hypothesis was observed using Bayesian analysis. This was somewhat surprising, given that autistic adults did perform the same task less accurately than neurotypical adults, but this result demonstrates that difficulty making line-of-sight judgements during face-to-face interactions likely does not contribute to social communication difficulties associated with the BAP.

An inherent limitation of face-to-face paradigms is that they do not afford the same level of experimental control as do more traditional lab-based computer tasks. Therefore, in the development of the current paradigm, care was taken to maintain control over extraneous factors that could have influenced performance and thus resulted in noise in the data. For example, the experimenters wore similar clothing for each testing session, wore minimal make-up and kept hairstyles very similar throughout. Testing always took place within the same testing room for each study and a consistent background visual array was always present. Moreover, the distance between the experimenter and each participant was made equivalent across each testing session by ensuring that the chairs remained a set distance apart, the participant was asked to ensure that their back remained in contact with the back of the chair throughout and the experimenter maintained the same position throughout the testing session. Finally, in an attempt to counter any subtle differences in task administration that could have arisen in response to the participant demographics, the experimenter in Study 1 was blind to the study hypotheses and the experimenters for Study 2 were blind to the participants’ BAPQ scores, as these were coded after the testing period. However, we acknowledge that the experimenter for Study 1 was not blind to the participants’ diagnoses, and hence it is possible that, despite the best efforts, there could have been some subtle differences between groups in the nature of the gaze cues presented, which is a limitation of this study design. It is recommended that other researchers conducting naturalistic social attention research also consider such factors in their experimental set-up, thus minimising between-participant differences that may influence data.

In conclusion, the current studies demonstrate that autistic adults are able to effectively follow the line-of-sight of a social partner during a face-to-face interaction. However, at the group level, overall accuracy is reduced indicating that making line-of-sight judgements is, at least, somewhat challenging for most autistic adults. Furthermore, it was clearly demonstrated that the overall level of autistic traits in neurotypical individuals did not predict accuracy of performance, indicating that the ability to make line-of-sight judgements does not contribute to social communication difficulties often observed in neurotypical individuals who are high in autistic traits. Overall, the findings presented contribute to furthering understanding of the mechanistic processes of social communication.

## Notes

1. The prior for the alternative hypothesis was equal to 1 since we did not make an a priori prediction of the correlation coefficient. Bayesian analyses were conducted in JASP version 0.8.4.
